# The relationship between GPs and hospital consultants and the implications for patient care: a qualitative study

**DOI:** 10.1186/s12875-016-0442-y

**Published:** 2016-04-14

**Authors:** Rod Sampson, Rosaline Barbour, Philip Wilson

**Affiliations:** Cairn Medical Practice, 15 Culduthel Road, Inverness, IV2 4AG Scotland; The Open University, Walton Hall, Milton Keynes, Buckinghamshire MK7 6AA England; Centre for Rural Health, The Centre for Health Science, University of Aberdeen, Old Perth Road, Inverness, IV2 3JH Scotland

**Keywords:** Interface, Primary healthcare, Secondary care, Relationship, Patient care

## Abstract

**Background:**

Improving the quality of care of at the medical primary-secondary care interface is both a national and a wider concern. In a qualitative exploration of clinicians’ relationship at the interface, we want to study how both GPs and hospital specialists regard and behave towards each other and how this may influence patient care.

**Method:**

A qualitative interview study was carried out in primary and secondary care centres in NHS Highland health board area, Scotland. Eligible clinicians (general practitioners and hospital specialists) were invited to take part in a semi-structured interview to explore the implications of interface relationships upon patient care. A standard thematic analysis was used, involving an iterative process based on grounded theory.

**Results:**

Key themes that emerged for clinicians included communication (the importance of accessing and listening to one another, and the transfer of soft intelligence), conduct (referring to perceived inappropriate transfer of workload at the interface, and resistance to this transfer), relationships (between interface clinicians and between clinicians and their patients), and unrealistic expectations (clinicians expressing idealistic hopes of what their colleagues at the other interface could achieve).

**Conclusion:**

The relationship between primary and secondary care clinicians, and, in particular, difficulties and misunderstandings can have an influence upon patient care. Addressing key areas identified in the study may help to improve interface relationships and benefit patient care.

**Electronic supplementary material:**

The online version of this article (doi:10.1186/s12875-016-0442-y) contains supplementary material, which is available to authorized users.

## Background

Historically, the relationship between general practitioners (GPs or ‘family doctors’) and specialists has been uncomfortable, partly because of the hitherto relatively low status of GPs [1]. Despite the United Kingdom National Health Service having transformed itself as a result of political pressures, patient demands, and advances in medical knowledge, both primary and secondary care services are essential for the system to ‘work’ [1–3].

Many conditions are managed exclusively at the first point of contact in primary care, though some require more specialised medical attention necessitating transit across the primary-secondary care interface, usually to hospitals as inpatients or outpatients [4, 5]. For these patients, coordination between the different disciplines is essential for the delivery of quality care [6]. In countries with primary care services, effective communication and functional relationships across the interface are vital for both the delivery of optimal patient care, and the minimisation of risk to patient safety (e.g., in relation to significant changes in patient medications as they transition between primary and secondary care [7–9]) [10–13], especially since primary and secondary care clinicians can act as separate ‘professional tribes’ [14]. Integration (incorporating relationships between people which need to be developed if integration is to be meaningful and sustained) of primary-secondary care services is dependent in part on interface team working, and effective communication between the two [15, 16]. There is evidence that most clinicians work hard, sometimes over many years, at developing good personal relationships with their colleagues based partly on the perceived benefits for the patient of a relationship based on trust and mutual respect [1].

Manifestations of the primary–secondary care interface across the world are diverse, but there is evidence of common ground in most healthcare systems. [17–19] In countries where general practice (or family medicine) is well developed, there are similarities in the functions and characteristics of the primary–secondary care interface-based system, with GPs usually acting as ‘gatekeepers’ to secondary care to some extent [5, 17]. This is the context of the interface in Scotland (United Kingdom) where our study is based.

In countries with ‘gatekeeping’ primary care systems, there has been an increased focus on the interface between primary and secondary care [20–25], highlighting the importance of better relationships between hospital and community, and between specialist and GP [5]. Qualitative synthesis of patient experience at the interface has further confirmed the importance of relationships between clinicians, with a recommendation that the influence of these relationships upon patient care be explored further [26]. Developing relationships between GPs and specialists may motivate increased collaboration, and coming to better know one another may improve the quality of collaboration [27]. Improving the quality of care of at the medical primary-secondary care interface is both a national and a wider concern [28–30].

Research suggests that the nature of clinicians’ connection at the interface, and how they regard and behave towards each other, may influence patient care [26]. However, very little qualitative research has been carried out with regard to these relationships and it is in this context that we designed a study to explore the relational perspectives of both GPs and hospital specialists.

## Methods

The study was conducted in NHS Highland between August and December 2014, and involved semi-structured interviews with a purposive sample of clinicians [31], selected to reflect both the different contexts in which care is provided (those clinicians working in urban and rural areas [32, 33]); and clinicians’ characteristics (gender). Since specific respondent details may compromise confidentiality in a small cohort such as this, characteristics such as age, number of years in practice, ethnicity and country of graduation have not been provided.

Clinician details (name, gender, speciality, location) were obtained [34, 35], and those meeting eligibility criteria (Appendix 1) were entered into a sampling grid (Table 1), and then allocated a sequential number. Using Excel Random Number generator, clinicians from each cell were selected, and then sent an invitation. Clinicians agreeing to take part were consented using a standard form (Additional file 1), and offered either a telephone or face-to-face interview. The selection process continued until all cells were represented in the sample [36]. We sought to include equal numbers of men and women, from primary and secondary care, in urban and rural locations.

**Table 1:** Numbers and characteristics of those agreeing to interview, and those not responding to invitation

Those responding and agreeing to being interviewed*;				
Gender	Primary care		Secondary care **	
	Urban	Rural	Urban	Rural
Male	3	2	5	3
Female	3	2	2	2
Those not responding to invite;				
Gender	Primary care		Secondary care	
	Urban	Rural	Urban	Rural
Male		4	1	3
Female		4	4	3

*No invited clinicians responded to say they would not like to be interviewed

**Specialties represented included Emergency Medicine, General Medicine, General Surgery, Neurology, Ophthalmology, Paediatrics and Psychiatry

The topics to be covered in the interview schedule were developed in collaboration with two GPs and three specialists, and these schedules were then piloted and further refinements made. Interviews were carried out (RS) using the final interview schedule (Appendix 2), and were audio-recorded, transcribed and entered into nVivo 10.0 software in preparation for analysis.

Thematic analysis of transcripts was carried out, and categories and codes were further developed and interrogated, following an iterative process broadly based on grounded theory [37]. Analysis of individual transcripts by RS commenced as the interviews progressed, with open coding gradually being built into broader categories, whereby higher-level recurring themes were identified and sub categories developed and refined. Discrepancies in coding were resolved by discussion between the authors (RS/RB/PW), in order to ensure consistency and discussions allowed the team to capitalise on different disciplinary insights [32]. In particular, the analysis sought to draw on participants’ concepts (i.e., ‘in-vivo codes’) [37]. Constant comparison allowed for identification and exploration of patterning in the data.

The study gained University ethical approval (Aberdeen University), and NHS R&D Management approval.

## Results

A total of 41 clinicians were invited, and 22 agreed to take part in a semi-structured interview (Table 1).

Participants’ responses can usefully be grouped into four main themes; communication, conduct, relationships, and unrealistic expectations.

### Communication

Communication was an important theme for clinicians at the interface, with subthemes of accessing and Ping-Pong, listening, and soft intelligence described. Communication was necessary in a number of interface contexts including where a GP was referring a patient to secondary care (either to outpatient clinic or as an emergency), where a specialist was discharging a patient to primary care (e.g., from outpatient clinic or as in inpatient), or where GPs and specialists simply needed to communicate with one another about specific aspects of patient care.

#### Accessing and Ping-Pong

Clinicians expressed problems with accessing one another; having to go “back and fore.” This varied between departments (for GPs) and practices (for specialists). Contextually, for GPs this may have been in relation to seeking specialist advice regarding a patient; for a specialist, this may have been in relation to trying to contact a GP to communicate a significant new diagnosis in a patient. This “Ping-Pong” type of communication also existed where letters transitioned the interface, as each side tried to set boundary lines of responsibility, and was more apparent where a perceived lack of respect between clinicians, and poor working relationships between individual specialists/specialities and GPs/GP practices. Clinicians on both sides of the interface raised this issue-as evidenced by comments from, firstly, a hospital consultant and, secondly, a GP:

**Table:** 

HRF47*; I’m very aware that 98 % of the time I won’t get hold of the GP that I want to at that point, then I’ve got to remember to phone him back later which, to be honest, is quite bizarre. *[Nomenclature; Setting/Geography/Gender/Participant number. So for example, GPUF3 indicates a female GP in an urban setting, participant number 3. HRM4 indicates a male hospital specialist in a rural setting, participant number 4.]

**Table:** 

*GPUF6; I think there are certain departments where you just think if you try and get, try and speak to a consultant you either won’t manage or it will be difficult or it will take lots of time and lots of kind of telephone ping pong.*	

A lack of understanding of each other’s working patterns could result in frustration for clinicians in terms of accessibility:

**Table:** 

*HUM14; “unfortunately being in a position with a phone with case notes and actually being able to get hold of someone at lunchtime, that’s one of the things that really pisses me off is when I phone and there is an automated message, the GP practice is now closed please phone back at 1.45 or whatever and unfortunately […] I think its years since I’ve been to the canteen for lunch”.*	

Hence, for this hospital specialist, this conveyed a sense that the GP is unhelpfully not available. However, one of the GPs suggested that specialists might be operating under misconceptions about GPs and their availability:

**Table:** 

*GPRF34;” I think they think we are difficult to get hold of, I think they think that we are all part time and stuff and that you know the GP is not there again. The GP is not there again because they are maybe out on visits or they are in surgery […] then they leave a message and you phone them back or you do this kind of ping pong which is intensely frustrating for everybody.”*	

#### Listening

A sense, for the GP, of being listened to at the point of urgent hospital referral (or when seeking specialist advice) gave an impression of working together for the patient’s benefit. There was a sense for both GPs and specialists that knowing one another led to a “better conversation.”

Conversely, some GPs provided examples of poor listening as evidenced by the following quote:

**Table:** 

*GPUM3; “the whole conversation from start to finish felt like the doctor on the other end of the phone was trying to find a way of getting out of the conversation rather than us having a conversation to help a patient, it felt very much like he was trying to find a way that it wasn’t his to deal with, he kept interrupting my explanations, the effect of that would be that he kept asking questions that I had already given him the information if he had just listened to me.”*	

Likewise, in complex community-based patient scenarios that did not readily fit secondary care systems/guidelines/protocols, listening to one another appeared to be even more important in relation to patient care, as described by this rural GP:

**Table:** 

*GPRF10; I’m just not trying to tick boxes you know what I mean, I know we could get her scoped you know I know that but listen to me I’m trying to tell you something else, I’m trying to say that you know she is frail, I’m wanting you to think a little bit more holistically than that, I want you to you know, I know you can scope people, and I don’t even want you to scope her I just wanted you to think a little bit more kind of, I want you to support me making these very difficult decisions cause sometimes I feel I am on my own the whole time.*	

#### Soft intelligence

“Soft intelligence” (an ‘in-vivo’ code, i.e., a phrase taken directly from the data referring to the supply of relevant psychosocial details in the written referral letter when a GP referred to specialist outpatient clinic) was seemingly essential for specialists, and its communication appeared more likely in a proven and established relationship, providing important context for a patient referral:

**Table:** 

*HUM14; “some individuals will write factually a very good letter but they don’t actually give you a bit of the flavour of the patient […] you know that the patient’s husband died last year and she is really very anxious, speaks volumes”.*	

In some cases, communication of “soft intelligence” had a specific impact on care provision e.g., the patient being allocated a “double” outpatient clinic appointment. Conversely, the lack of “soft intelligence,” could lead to unnecessary patient investigation, and an increase in patient anxiety.

**Table:** 

*HUM13; “we have all this referral pathway forms which is just […] a kind of magic tick box exercise and we often get referrals from GPs that there is no detail […] there is a couple of tick boxes and because they fit the criteria it gets them across along the urgent referral pathway cancer but there is no attention about, there is no personalised history, you know, what has happened to the change in bowel habit, you know is the patient single, do they live at home, you know what medications he takes, we don’t get that, we just get patient has got worrying symptoms and has to go along the pathway […] so that is better for the patient who generally has got cancer but for the patient who hasn’t you know you create anxious on the patient side but it also doesn’t allow any background issues to manufacture itself across from the referral.”*	

### Conduct

This theme centred on perceptions of interface colleague’s professional behaviour: clinicians dumping (defined as an inappropriate transfer of workload across the interface) and resisting (a term used to depict the opposition of colleagues to take on work being handed over to them).

#### Dumping

This was an issue for both GPs and specialists, but for each, in relation to differing aspects of patient care. For specialists, dumping referred to a GP’s inactivity in an area of care previously perceived as obligatory (e.g., “out of hours,” in the provision of continuing care, or in the specific clinical management of a condition), which was then felt to result in additional workload for secondary care. Some specialists suggested that in some instances (e.g., the management of childhood constipation), practices may have managed a patient “in-house,” but were now more likely to refer to secondary care. The following specialist expressed dissatisfaction in GP colleagues in this regard, seeing “out of hours” care as an essential part of working as a healthcare professional:

**Table:** 

*HUM39; “I have perception that there’s huge pressures, […] it’s a hard job (being a GP) so I can imagine most people would lack energy to contemplate doing out of hours but at the same time I suppose we all have negative parts of the job we have to take on and I mean I don’t want to do on call but I accept that’s a part I’m going to do until I retire”.*	

For GPs, in contrast, dumping was related to perceived work transfer (from secondary to primary care) without discussion or subsequent resource reallocation. This work transfer included, for example, responsibility for following up results of secondary care initiated investigation, and the follow-up of patients in the community (where there was a perception of patients being discharged too soon from hospital). GPs alluded to a lack of control over further elements of workload that were apparently being transferred to them on a regular basis. Some GPs, as evidenced by the following quote, did not readily understand reasons behind this apparent transfer:

**Table:** 

*GPUF4;” I personally feel that we are being dumped upon because other colleagues are not completing their job you know, why are they doing that? Are they doing that because their training wasn’t sufficient? Are they doing that because they are lazy? Are they doing that because they are too busy? We could all say we are too busy”.*	

Others, however, expressed the view that specialists were simply not accepting a level of professional responsibility:

**Table:** 

*GPUM1;”well that’s when you know the usual games, patient phones up [hospital], speaks to secretary saying I need to be seen sooner, the secretary speaks to the consultant, consultant says tell them to go through their GP, the patient comes to me, […] I write to the consultant and the reply comes back “which one of my patients do you want me to budge out the way so that your patient gets in first?” So that’s the sort of nonsense that I would like to get rid of and for, it’s the hiding behind organisations, hiding behind waiting lists, you know it’s the culture, if somebody phones up and says I need a home visit today they get one or they get seen, somebody phones a consultant, I write to the consultant saying this patient needs seen, the consultant has no obligation whatsoever to see the patient. It’s that lack of ownership of demand, it’s for consultants feeling the pressure and feeling the demand and the clinical need as much as we do. That’s what I’d like them to share in.”*	

Specialists and GPs were thus, in essence, voicing similar concerns; a sense that the “other” wasn't fulfilling their professional responsibilities, leading to a loss of goodwill and a disappointment in one another.

#### Resisting

Specialists were alone in describing a degree of “resistance;” more evident where poor interface relationships with individual GPs or practices existed. This “resistance” impacted negatively on shared care, leading to a sense of not “being in it together.” From their perspective, specialists described working hard to maintain relationships, for the benefit of patient care. It is worthy of note that specialists used the word “sharing” of care (in comparison to the notion of “transfer” referred to by GPs):

**Table:** 

*HRF47; “what I’ve noticed is everybody is less willing to go the extra mile and I think that accounts for both secondary and primary care and it’s a direct reflection on other pressures that are being put on the system […] the issues about physical health checks for psychiatric patients and all the other things that secondary care have traditionally asked primary care to do, […] its now being met with a degree of resistance and […] I think that’s led to a real deterioration for shared care and patient care as a whole.”*	

### Relationships

This theme focussed on the professional relationships between GPs and specialists, and in the case of continuing care, between clinicians and their patients. Different types of relationship were described including proven social relationships with professional interaction, and those purely built on professional grounds. Working relationships may have been developed through mutual correspondence (written or phone) over the years, without the specific clinicians actually having met one another. Clearly, some relationships were positive in terms of interaction and in terms of shared care of patients, while for others, the converse was true.

#### Continuing care

Continuity of care is defined as a continuous relationship between a patient and an identified health-care professional who is the sole source of care and information for the patient. Clinicians on both sides of the interface felt that such continuity of care had changed due to increasing doctor numbers (in the context of subspecialisation and part-time working), mobility of the workforce (less likelihood of remaining in a post long term), and reliance on locum use.

Specialists sensed reduced continuity within primary care leading to a felt need to fill a “continuity gap” previously unoccupied by them:

**Table:** 

*HUF40; I think the days where a patient has one GP who knew them from cradle to grave are gone, patients will often say you know I see a different GP every time I go to the practice.*	

Specialists described short-term locum use in rural practices having a negative impact upon patient care; the lack of a consistency limited the specialists’ ability to deliver routine care (including treatment delays, unimplemented guidance, or unnecessary patient journeys to hospital). Conversely, practices making use of “regular” locums, where interface relationships could be established, minimised this effect:

**Table:** 

*HUF17; there are some practices where there isn’t a permanent GP at the moment […] and I have had problems there where patients have clearly lacked the support of a good GP and then the things that I suggested haven’t happened, I think that relates to a lack of a service in certain practices.*	

#### Implications for patient care

Quality of clinician relationship influenced patient care at the interface. While most felt professional relationships shouldn't impact on patient care, they gave examples of how relationships did impact upon patient care. For GPs, good personal relationships with specific specialists led to a sense of being better supported with patient management, of facilitating direct communication with specialists, and of easing patient transitions (“smoothing the waters” as one clinician described it).

**Table:** 

*GPUM1; Consultants that I know that I have met face to face tend to give quicker and more comprehensive responses regarding referrals that I make either by [electronic hospital referral software] or by email, so knowing them personally makes a huge difference. They write more pleasant letters and are more helpful. Consultants that I don’t know are more distant and tend to give more hand offs and bounce people about rather than give a helpful response.*	

Specialists described their trust in, and perception of a GP’s competence, as impacting upon patient care. They identified that similar referral paragraphs from different GPs, could lead to different responses (and therefore outcomes for the patient) based on their prior knowledge and experience of the referring GP. A good personal relationship facilitated a sense of wanting to help one another (rather than perceiving contact as a burden) and assisted the ease with which specialist advice would be given, and be accepted by GPs.

Having personally met their GP colleague was acknowledged as influential in how they may respond to a request.

**Table:** 

*HUM14; 2 people can write a paragraph which is exactly the same thing and depending on who it’s come from I personally put on a lot of different weight on it.*	

#### Forming relationships

Clinicians listed a number of facilitators to forming relationships, including the use of shared space, the role of education, and purposefully modifying their tone of communication when liaising with interface colleagues.

Exclusive to rural GPs was the experience of being able to work closely alongside visiting specialists, which led to them e.g., being able to “sit in” on specialist clinics involving their own patients. The familiarity of one another, in this shared space, allowed GP managed patients to have better access to specialist input (compared with more conventional routes) as evidenced by this quote:

**Table:** 

*GPRM31; “we just nip upstairs and ask them if they will see somebody on the ward who is concerning us if it happens to be within their specialty or we’ll write ahead and ask while you’re here would you please come and see somebody on the ward and so on, so we get to know them […] we see them face to face, they see us and they put a face to the name on a letter and nobody has measured it but I would say that for GPs who run the community hospital where the out patient clinics are that there’s a, I would say a much better sense of communication and team spirit between GP and consultant than our colleagues who don’t work in community hospital”.*	

Medical education was felt to be a method of developing interface relationships. A common awareness for older clinicians was that joint educational events were less evident. Specialists were more vocal (than GPs) in aspiring to resurrect such joint events, and were uniformly sensitive to suggest that such events should involve shared learning (compared with a more traditional didactic approach). Those organising joint events, described the “same cohort” attending, leaving those not attending remaining less engaged. The timing of such educational events was important, with some less likely to be motivated to attend if organised as an evening meeting at the end of a long clinical day.

**Table:** 

HUM39; so that’s something we link a PLT* afternoon with a hospital audit afternoon and actually get people together through some kind of educational programme, and the education programme doesn’t have to be particularly important its more a bit of group work, which is 6 folk round a table, 3 secondary care, 3 GPs and what your actually talking about is immaterial for the large part, it just means I met you in that PLT afternoon whatever so maybe we’re just a bit too in “silos**” for post graduate approach.	

* “PLT”-“Practice Learning time”-a session of time where a group of primary care practices close during working hours to pursue learning needs.

**”silos” -a description of groups of people (in this case interface clinicians) working in isolation to one another.

Barriers to forming relationships were described, with increasing doctor numbers impacting negatively on the ability to establish connections. GPs underlined a lack of information when a new consultant arrived in the area. Specialists highlighted a greater proportion of part-time working, and locum use, as providing difficulty in keeping track of who was who in primary care. Clinicians on both sides acknowledged excessive workload as a barrier to developing relationships.

**Table:** 

*HUM13; in the past there used to be, there was a lot more involvement between primary care and secondary care but you know all the consultants in hospitals knew all the GPs but I don’t think that is possible any more I just think the population is expanding, the health care is becoming so complex, everybody is becoming so sub specialised, […] and likewise in primary care you know these days you get GP practices which are expanding, you are getting a lot of locums coming in if you can’t find the right people, there is a constant flux of people, I think it’s very difficult that personalised relationship I think it’s ideal but I don’t think it’s possible anymore, it’s difficult.*	

*Working in each other’s kingdom* (another ‘in-vivo’ code) referred to the perceived benefit of spending time in each other’s workplaces, to better understand each other’s roles. GPs were keen to spend time in hospitals, in order to improve their knowledge of colleagues, and their work. GPs, however, also wanted specialists to spend part of their training in primary care, to encourage both a greater understanding of the patient in the community, and a greater appreciation of the GP role.

**Table:** 

*GPUM3; I do think it would be useful for everyone in their training to spend some time in primary care.*	

### Unrealistic expectations

Clinicians described their interface colleagues as sometimes being guilty of creating unrealistic expectations in patients of what could be achieved in primary or secondary care.

For GPs, unrealistic expectations centred on patients being misled as to when secondary care test results would become available (to GP or patient), or in the timing of specialist outpatient follow-up. This led to patients booking appointments in primary care only to be told “the result isn’t back yet,” which was felt to waste both doctor and patient time. In regard to timing of outpatient follow-up, GPs described patients being misinformed at a number of levels (either directly by the specialist at clinic, by the nurse on exiting the specialist clinic, by the specialist secretary, or secondary care appointments administrator) leading to anxiety on the part of the patient (when an appointment didn't come through in the expected timescale), and time spent in GP consultations, trying to explain to the patient why such misinformation may have occurred:

**Table:** 

*GPRF34; “she saw the consultant last September and was told that she would be reviewed in 3 months time, […] eventually this month she came back to see me again saying that it’s a year since and I was still seeing her every 3 months to make sure there is no obvious clinical recurrence and when we phoned up this time I actually phoned up the secretary rather than my secretary phoning up and said what’s going on and they say she is on the list, […] we are running 11 months behind. I said to them why am I only finding that out now cause actually you would have known, the consultant should have known last year when he said see you in 3 months knowing he wouldn’t see her for 11 months and its to do with the secretary said well the consultants choice is that she is seen in 3 months and its the hospital who cant fulfil that, it’s completely bats, its mad.”*	

In context, GPs described frustration with both patients and themselves being left in a “limbo of uncertainty.” Uncertainty was also felt by GPs at being left to communicate results of specialist investigations to patients (an unrealistic expectation that the GP would know the implications of certain test results, and any subsequent necessary follow-up).

For hospital specialists, unrealistic expectations were related to what GPs wrongly assumed was possible in hospital, or in GPs’ failure to understand the difficult working environment under which specialists worked.

Clinicians acknowledged that understanding one another more, and the context in which each other worked, may help reduce such unrealistic expectations.

Summaries of the main themes generated from clinician interviews are outlined in Table 2.

**Table 2:** Summaries of the main themes generated from clinician interviews

Theme	Sub-theme	Additional sub-theme
Communication	Accessing and Ping-Pong	
	Listening	Good listening impacting positively on patient
		Poor listening impacting negatively on patient
	“Soft intelligence”	Provision of, depends on quality of relationship
		Certain clinicians better able to pick up upon
		Is valued by Hospital specialists
Conduct	Dumping	Abdication of responsibility
		Has increased over time
		Hiding behind an organisation
	Resisting	
Relationships	Continuing care	
	Implications for patient care	Good relationships benefit patients; smoothing the water and going the extra mile.
	Forming	Facilitators
		Barriers
		Working in each others Kingdom
Unrealistic expectations	Creating them in patients	
	Of each other	

## Discussion

### Summary of findings and comparison with existing literature

In this qualitative study focusing on the relationship between primary and secondary care clinicians, we found areas of shared concern (e.g., difficulties in accessing and communicating with one another, inappropriate transfer of workload across the interface, and creation of unrealistic expectations), areas of more importance to primary care (e.g., the need to experience one another’s work environments), and areas of greater meaning to secondary care (e.g., communication of soft intelligence, and reduced continuity within primary care). Facilitators to developing relationships were acknowledged (e.g., meeting with one another in an educational context), and barriers described (e.g., excessive workload).

#### Communication

Communication problems described in our study have, elsewhere, contributed to fragmentation of patient care [11–13, 26]. Clinicians expressed annoyance with problems associated accessing one another, and, in the absence of a “universal pause (i.e., a shared time where clinicians were known to be accessible)” expressed the feeling of being caught up in communication “Ping-Pong”. In our analysis, communication problems were identified as an issue for clinicians on both sides of the interface (leading to both themselves and the patient being left in a “limbo of uncertainty”), which contrasts with previous research [38] where GPs alone described inadequate specialist communication. Soft intelligence, in a healthcare management context, is a term “associated with seeking and interpreting soft data of the kind that evade easy capture, straightforward classification and simple quantification to produce forms of knowledge that can provide the basis for intervention.” [39] In our study, specialists used the term to describe the communication of relevant psychosocial detail by GPs in referral letters, which was more likely to happen in the context of an already established relationship, and, where this did not occur, had the potential to generate unnecessary patient investigation and anxiety.

#### Conduct

Clinicians described increasing dumping across the interface. For specialists, this in relation to what were perceived as inappropriate referrals, or GPs not meeting their obligations (e.g., providing an “out of hours” service). For GPs dumping related to what was seen as uncontrolled transfer of work, unaccompanied by resource reallocation. Both for GPs and specialists, a perceived 'abdication of responsibility' led to disappointment with each other. It is noteworthy that for clinicians in our study, at a time where it is recognised that successful interface working is reliant on integration and collaboration based on positive working relationships, that there might be a sense of increasing concern around conduct eroding such interaction [15, 16, 27].

#### Relationships

Specialists expressed the view that decreasing continuity within primary care (a view also expressed by patients elsewhere [40]) led to them feeling responsible for making good this “continuity gap.” This lack of continuity was described not only in relation to the doctor-patient, but also in relation to the clinicians’ workings across the interface divide. There is agreement that, at boundaries and interfaces, continuity of patient care is essential across its three core dimensions; informational, management and relationship continuity [6, 41, 42]. In relation to the latter category, team continuity was identified in our study as an important, but frequently overlooked dimension. In order for informational and management continuity to operate well at the interface for the patient, both primary and secondary care teams need to be helped to see they are working as one larger team (which will incorporate trust of one another, communication with each other, and agreeing a clear communicated plan clarifying longitudinal lines of responsibility) [43]. In our research, continuity of care may be seen to be limited in some instances by interactions between people who don’t know one another, don't recognise themselves as part of a larger team, and who seem to have an adversarial, disrespectful or distrustful relationship with interface colleagues.

Historical studies of the profession have highlighted problems with the way GPs and specialists relate to one another [44, 45]. Patients have also reported such tensions in relationships between clinicians [26, 46]. The importance of developing relationships at the interface based on trust highlighted in our study should not be underestimated, and may be essential for effective harmonisation of care; “cooperation is a by-product of trust […] rather than a source of trust” [47]. Indeed, moving towards a more co-operative patient centred approach will require deep-rooted relational and organisational conflicts to be replaced by more co-operative alliances [48]. Fukuyama notes that developing such trusting relationships is a part of an organisation’s social capital, i.e., interface clinician’ willingness and ability to come together for the benefit of patient care [49–51]. Innovative initiatives, aimed at fostering and maintaining such social capital are already leading to benefits in terms of working relationships at the interface, with a future hope of “improved patient access, enhanced patient pathways and great patient experiences” [52]. Horder stipulated a set of conditions that require to be fulfilled in order to develop such relationships, including meeting with one another; “bad relationships thrive on isolation [53].” Specialists were keen to stress that joint educational events with GPs should reflect “shared learning” and not duplicate the traditional hierarchical process; this contrasts with previous research with specialists feeling they had little to learn from GPs [1, 54]. Joint learning events might also help clinicians to develop networks and share learning as a means of establishing “Communities of Practice” [55].

Rural GPs, in contrast to their urban peers, appreciated having the opportunity to work closely alongside visiting specialists. Earlier investigation however has hinted at the limited benefit of such arrangements [54, 56, 57].

Clinicians on both sides of the divide highlighted excessive workload, and increasing doctor numbers, as presenting barriers to forming good relationships.

#### Unrealistic expectations

Clinicians at the interface described creation of unrealistic expectations in patients, and of each other. More accurate information (e.g., in relation to timing of test results, or of wait for specialist appointment) being provided to the patient may help reduce frustration (for patient and GP) when it becomes apparent that suggested timescales were predictably unachievable. Clinicians acknowledged that understanding one another more, and the context in which each other worked, may help reduce unrealistic expectations of each other. Of interest, despite hospital specialists bemoaning GPs’ failure to understand the difficult working environment under which specialists worked, the aspiration of “working in each others kingdoms (e.g., time spent “shadowing” the other for a day to better understand a colleagues work)” was singularly suggested by GPs, but not by specialists (also a finding in previous research) [27].

### Strengths and limitations

The research team sought to ensure that a range of demographic and professional perspectives were included within the interview sample, which means that the ensuing sample reflected diversity rather than being representative [58, 59]. While the study was located in one health board area, both the broad spectrum of participants and the structural similarity of the primary/secondary care interface to that of other regions provides grounds for transferability of findings. NHS Highland is represented well with rural practitioners; this study therefore affords insights that may be valuable for other areas with clinicians based in rural areas. It is noteworthy that all urban GPs approached took up the offer of invitation. This may well have been influenced by the position of the interviewer as an urban GP working in the study area. Reasons for non-response to study invitation were not sought, and it is not known therefore to what extent differences between those who did/didn't respond would impact upon the generalisability and transferability of study findings.

## Conclusion

Our findings suggest that interface relationships between GPs and specialists are important in terms of the potential to influence patient care. Addressing barriers to forming relationships (including greater clinician workloads and less shared meeting time) would seem necessary in order to improve effective care delivery. New methods of sharing information across the interface where a new clinician arrives in an area may be usefully established. National and local health authorities may consider the merits of establishing a “universal pause.” Of note was the unique experience rural practitioners had in working closely with visiting specialists (facilitating relationships, and allowing unique patient access to specialists); there may well be merit in exploring the potential for greater use of shared space involving GPs and specialists, in both urban and rural areas.

Efforts to promote the sense (and necessity) for primary and secondary care groups to see themselves as part of a larger team (promoting continuity of patient care across the interface) would seem essential; inherent in this would be acknowledgement of the importance of building trust between interface clinicians, to the ultimate benefit of patient care. Investing in the social capital of interface relationships may be helpful here. One specific recommendation that may be considered is the formal promotion of “shadowing days” where GPs and specialists spend time in each others “kingdoms,” to help them to better understand each others roles, minimising the generation of “unrealistic expectations.” Such practice may usefully form a mandatory component of continuous professional development. Speciality colleges may consider how all trainees might experience working in both primary and secondary care. Initiatives promoting co-mentoring between interface clinicians may also be a novel approach to consider.

Joint working groups (involving clinicians from both sides of the interface) to help formulate shared clinical guidance, analyse where things could have gone better, or work together through dumping/resistance issues, may help foster a sense of “we’re in this together.” Such groups will only be effective with support from national and local heath care managers.

Clinicians saw education as a tool for developing relationships, with specialists keen to emphasise a model of shared learning. Key decision makers may wish to consider how best this may be delivered in the midst of system constraints. Aligning primary and secondary care protected learning times may be advantageous, given “out-of-hours” educational meetings may be limited in their ability to engage with clinicians who have worked an intense clinical day.

There were clear examples of how good communication across the interface could positively influence patient care. Presently, there are no standardised approaches to communication across the interface (or shared knowledge on accessibility and preferred method of communication for individual clinicians); this may be an important area of focus, both for future research, and for high-level policy consideration. Embracing newer technologies to assist in this (e.g., email, video-conferencing, online communication) may be of value.

It is encouraging to note that despite acknowledged divisions between primary and secondary care, there exists a will and determination within clinicians working at the interface to improve things for both themselves and for the benefit of patient care.

## Appendix 1

### Study population & eligibility criteria

#### Inclusion criteria

1. All GP Partners in NHS Highland who are active in their roles at time of study commencement.
2. All Hospital Specialists at Consultant level in NHS Highland who are active in their roles at time of study commencement and who;
  - Are involved in delivering care to patients referred by their GP to out patient clinics, and,
  - Are involved in delivering care to patients referred by their GP to hospital for inpatient care.

Specialities meeting inclusion criteria include Accident & Emergency, Care of the Elderly, Chest Medicine, Clinical Oncology, Endocrinology, ENT, General Medicine, Haematology, Obstetrics & Gynaecology, Ophthalmology, Oral Surgery, Orthopaedic Surgery, Paediatrics, Palliative Care medicine, Adult Psychiatry, Psychiatry (Child & Adolescent), Rehabilitation Surgery, and Urology.

3. Good understanding of written and spoken English, not requiring an interpreter to understand the details of the study or to complete the paperwork required.

#### Exclusion Criteria*

1. All GP’s** in NHS Highland who are not partners (this includes for example GP Speciality trainees and salaried Doctors) at time of study commencement.
2. All Hospital Specialists at Consultant level** in NHS Highland who are active in their roles at time of study commencement but who;
  Are not involved in delivering care to patients referred by their GP to outpatient clinics, or,
  Are not involved in delivering care to patients referred by their GP to hospital for emergency care.
3. Inadequate understanding of written and spoken English to understand the details of the study or to complete the paperwork required.

*Some hospital specialities in NHS Highland will include no clinicians meeting inclusion criteria because they do not see patients as outpatients and/or don’t deliver inpatient care (e.g., Anaesthetics, Bacteriology, Biochemistry, Breast Screening, Cytopathology, Nuclear Medicine, Occupational Health Medicine, Orthodontics, Community Paediatrics, Pathology, Radiology, Restorative dentistry, and Sexual Health), whilst other hospital specialities (e.g., Accident & Emergency, Care of the Elderly, Chest Medicine, Clinical Oncology, Endocrinology, ENT, General Medicine, Haematology, Obstetrics & Gynaecology, Ophthalmology, Oral Surgery, Orthopaedic Surgery, Paediatrics, Palliative Care medicine, Adult Psychiatry, Psychiatry (Child & Adolescent), Rehabilitation Surgery, and Urology) will include clinicians meeting criteria.

**Speciality trainees have been excluded since their training programmes may contain both primary and secondary care posts which change within the timescale of the study. Salaried and sessional clinicians (in either primary or secondary care) are excluded on the basis of their varied roles within the NHS potentially not providing a robust distinction between primary and secondary care in the context of our study aims.

## Appendix 2

1. How long have you worked in your clinician role? What size of practice or department do you work in (for GP’s this may be something about list size, for hospital consultants something about number of beds in the hospital for which they are responsible)?
2. What would you consider most important in terms of relationships with your primary/secondary care colleagues?
3. How has your relationship with your primary/secondary care colleagues impacted on patient care?
4. How would you describe your experience of the primary care/ secondary care interface?
  - How has it been for you and your patients?
  - How do you view your colleagues from the other sector of care? What really evokes strong thoughts or feelings about them?
  - How do you think they view you?
5. Give an example where the interface worked well. How did this impact on you or the patient?” Then follow with “Can you describe a specific time when you have felt that ‘interface issues’ impacted negatively on you, or your patient(s), i.e. describe a specific ‘interface problem’ from your perspective?”
  - What happened?
  - Were there communication issues? Tell me more.
  - Were there relationship issues? Tell me more.
  - How do interface issues play out in your day to day work? Examples?
6. What do you think could address the ‘interface issues’ that you have described (if, of course the participant has described any!) and as a result to improve patient care? Best case scenario? Worst case scenario?
  - How might you play a part in this? Consequences-positive and negative?
  - How might your colleagues in your sector play a part? Consequences-positive and negative?
  - How might your colleagues in the other sector play a part? Consequences-positive and negative?
  - How does this compare with other areas you have worked?
7. How do your colleagues view the interface? What is the range of experience you are aware of?
8. Have interface issues changed over time? How much control do you feel you have over some of these issues?
  - Contacts moving jobs/leaving the geographical area?
  - Political restructuring?
  - Patient demands?
  - Advances in medical knowledge?
9. What advice would you give to someone starting out on their professional career in the context of your relationship with your primary/secondary care colleagues?

## Additional file

Additional file 1: A Qualitative Exploration of the Relationship Between Primary and Secondary Care Clinicians. What would make a difference to Patient Care? (DOC 112 kb)

## Abbreviations

GP: general practitioner
NHS R&D: National Health Service Research and Development
