# Supplementary material for: The relationship between GPs and hospital consultants and the implications for patient care: a qualitative study
Source: BMC Fam Pract. 2016 Apr 14;17:45. doi: 10.1186/s12875-016-0442-y (PMC4831146; doi:10.1186/s12875-016-0442-y)
Supplement: Additional file 1: — A Qualitative Exploration of the Relationship Between Primary and Secondary Care Clinicians. What would make a difference to Patient Care? (DOC 112 kb) [file 12875_2016_442_MOESM1_ESM.doc]

Identification Number for this research:

**Title of Project:** A Qualitative Exploration of the Relationship Between Primary and Secondary Care Clinicians. What would make a difference to Patient Care?

**Chief Investigator**: Dr Rod Sampson

**Please initial box**

| 1 I confirm that I have read and understand the Research Participant Information Sheet (one-to-one interview) dated 25.6.2014 version 2.0 for the above study. I have had the opportunity to consider the information, ask questions and have had these answered satisfactorily. I understand that my participation is voluntary and that I am free to withdraw at any time, without giving any reason. | ⬜ |
| --- | --- |
| 2 I agree to take part in the above study and agree to being audio recorded. | ⬜ |
|  |  |
|  |  |
|  |  |
|  |  |

________________________ ________________ ____________________

Name of Study Participant Date Signature

_________________________ ________________ ____________________

Name of Person taking consent Date Signature

(if different from researcher)

_________________________ ________________ ____________________

Researcher Date Signature
